# Plasma-free amino acid profiles are predictors of cancer and diabetes development

**DOI:** 10.1038/nutd.2016.55

**Published:** 2017-03-13

**Authors:** X Bi, C J Henry

**Affiliations:** 1 Clinical Nutrition Research Centre (CNRC), Singapore Institute for Clinical Sciences (SICS), Agency for Science, Technology and Research (A*STAR), Singapore, Singapore; 2 Department of Biochemistry, Yong Loo Lin School of Medicine, National University of Singapore, Singapore, Singapore

## Abstract

Type 2 diabetes (T2D) and cancers are two major causes of morbidity and mortality worldwide. Nowadays, there is convincing evidence of positive associations between T2D and the incidence or prognosis of a wide spectrum of cancers, for example, breast, colon, liver and pancreas. Many observational studies suggest that certain medications used to treat hyperglycemia (or T2D) may affect cancer cells directly or indirectly. The potential mechanisms of the direct T2D cancer links have been hypothesized to be hyperinsulinemia, hyperglycemia and chronic inflammation; however, the metabolic pathways that lead to T2D and cancers still remain elusive. Plasma-free amino acid (PFAA) profiles have been highlighted in their associations with the risks of developing T2D and cancers in individuals with different ethnic groups and degree of obesity. The alterations of PFAAs might be predominately caused by the metabolic shift resulted from insulin resistance. The underlying mechanisms have not been fully elucidated, in particular whether the amino acids are contributing to these diseases development in a causal manner. This review addresses the molecular and clinical associations between PFAA alterations and both T2D and cancers, and interprets possible mechanisms involved. Revealing these interactions and mechanisms may improve our understanding of the complex pathogenesis of diabetes and cancers and improve their treatment strategies.

## Introduction

Diabetes mellitus (DM) and cancer are two severe chronic diseases with tremendous impact on global health. Epidemiologic studies have shown that several forms of cancers, such as liver, pancreas, endometrium, colorectal, breast and bladder, develop more frequently in patients with diabetes.^1, 2^ Diabetes (primarily type 2, T2D) and cancers share many common risk factors, for example, aging, physical inactivity, diet and obesity. The potential biologic links between these two diseases are yet incompletely understood but may involve insulin resistance.^3^

Insulin resistance, intertwined with hyperinsulinemia, has been suggested as one of the possible underlying mechanisms for the direct connection between T2D and cancers.^3^ T2D is typically preceded by hyperinsulinemia to maintain glucose homeostasis.^4^ Additionally, convincing evidence have suggested that hyperinsulinemia may affect the signaling pathways of insulin and insulin-like growth factor 1 (IGF-1) and thus facilitate cancer development and progression.^5^ The etiology of insulin resistance has been either focused on lipid-mediated mechanisms^6^ or the interplay with obesity, which induces metabolic abnormalities.^7^ The latter is partly reflected in the abnormal circulating levels of lipid, protein and other classes of metabolites. Among the numerous metabolites, amino acids may have potential as excellent disease biomarkers because they are involved in protein synthesis and as metabolic regulators.^8^ In 1969, hyperaminoacidemia, manifested by elevated plasma-free amino acids (PFAAs) including branched-chain amino acids (BCAAs), that is, valine (Val), leucine (Leu), isoleucine (Ile) and aromatic amino acids (AAAs), that is, tyrosine (Tyr) and phenylalanine (Phe) in obese subjects, was reported.^9^ Hyperaminoacidemia in obesity may be a manifestation of increased insulin resistance.

Insulin has long been recognized as the regulator of branched-chain alpha-keto acid dehydrogenase complex, an enzyme complex involved in BCAA catabolism.^10^ Insulin resistance has been found to reduce the enzymatic activity of branched-chain alpha-keto acid dehydrogenase complex and hence suppress BCAA catabolism. This is considered as the plausible etiology of increased BCAA levels in obesity and/or diabetes.^11^ Indeed, evidence is accumulating that there is positive association between insulin resistance and circulating concentrations of BCAAs.^12, 13, 14, 15^ In addition, insulin resistance was shown to be correlated with the alterations of several other PFAAs, including AAAs, alanine (Ala), proline (Pro) and glycine (Gly).^16^

In recent years, PFAA profiles were found to be significantly altered in patients with diabetes and/or cancers.^17, 18, 19, 20, 21^ Little is known about the mechanisms, particularly whether PFAAs are contributing to the development of these diseases in a causal manner. However, the altered PFAA profiling appears to provide a great diagnostic potential and could be a promising biomarker for understanding the etiology and pathogenesis of diabetes and cancers.^22^

## Altered PFAA profiles in cancer patients

Cancer cells require certain amino acids, for example, glutamine (Gln), Gly, aspartic acid (Asp) and serine (Ser), for DNA synthesis, building new blood vessels, and duplicating their entire protein contents.^23^ They also require amino acids for proteins synthesis. These proteins work as growth-promoting hormones or tumor growth factors.^24, 25^ The increase in the amino acid demand may thus lead to a lower availability of PFAAs in cancer patients.^26^ Table 1 summarizes twenty studies specifically addressed the alterations of circulating amino acid concentrations in different cancer patients.

Vissers *et al.*^27^ analyzed the PFAA concentrations in three types of cancer patients with different levels of weight loss, that is, breast cancer (without weight loss), colonic cancer (occasional weight loss) and pancreatic cancer (frequent weight loss). They found a significant decrease in arginine (Arg) levels, regardless of tumor types and stages, weight loss or body mass index. This finding suggested that decreased Arg availability was a specific feature of the presence of a malignant tumor. They also revealed that BCAA concentrations were lower in all cancer patients than in age- and sex-matched controls; whereas TAAs were lower only in pancreatic cancer patients. It should be noted that the alterations of PFAA levels depend on the stage and the type of cancers. The study conducted by Gu *et al.*^28^ examined the PFAA profiles in 56 patients with gastric cancer, 28 patients with breast cancer, 33 patients with thyroid cancer and 137 healthy controls which were age matched. It was found that histidine (His) level was significantly decreased in breast cancer patients. Levels of Ser, Ala, Val, lysine (Lys), His, BCAAs, and TAAs were significantly decreased in gastric cancer patients. However, the thyroid cancer patients had significantly increased levels of methionine (Met), Leu, Tyr and Lys (Table 1). Besides the different types of cancers, the variation of PFAA pattern of patients was due to the different disease stages. Most of the patients with breast cancer or thyroid cancer in this study were characterized as early stage, whereas 12 of gastric cancer patients were characterized as advanced stage (stage IV). This study also showed that Ala, Arg, Asp and cysteine (Cys) promoted the proliferation of breast cancer cells. Alternatively, Cys promoted the proliferation of gastric cancer cells, but Ala and glutamic acid (Glu) inhibited it. These results underscored the potential function of the assessment of tumor-related PFAA patterns to examine and diagnose various cancers.

Recently, AminoIndex Cancer Screening (AICS) technology was employed as a novel cancer risk calculation method for early stage cancer diagnosis.^21, 29, 30, 45, 46^ In order to build AICS, 19 amino acids including threonine (Thr), asparagine (Asn), Ser, Gln, Pro, Gly, Ala, citrulline (Cit), Val, Met, Ile, Leu, Tyr, Phe, His, tryptophan (Trp), ornithine (Orn), Lys and Arg, were measured and statistically analyzed. For the colorectal cancer risk calculation in one case report,^21^ plasma levels of Ser, Pro, Val, Met, Ile and Lys were used. The AICS score was found to be 8.3, which indicated that the patient had an ~10-fold-increased risk of cancer. When the patient underwent colonscopy, a 10-mm adenoma-like lesion in the ascending colon with partial carcinoma was observed. The early detection of carcinoma using AICS method allowed complete resection, suggesting that PFAA profiles may provide a fast and easy diagnostic tool for cancers. Another study conducted by Fukutake *et al.*^20^ used Ser, Asp, Ile, Ala, His and Trp as variables to calculate the pancreatic cancer risk and successfully discriminate patients with pancreatic cancer (*n*=360) from control subjects (*n*=8372). They also analyzed the levels of 19 amino acids and a significant increase in Ser level and significant decreases in the levels of Thr, Asn, Pro, Ala, Cit, Val, Met, Leu, Tyr, Phe, His, Trp, Lys and Arg were observed in pancreatic cancer patients. Several other studies with small sample size^37, 38^ reported similar decreases in circulating amino acid levels in pancreatic cancer patients, which was interpreted as a result of the enhanced usage of PFAAs in tumors. Another possibility for the decreased levels of amino acids was associated with malnutrition. Patients with pancreatic cancer are usually troubled by malnutrition due to exocrine pancreatic insufficiency (EPI).

However, some studies investigating amino acid levels in plasma or serum samples from patients with breast cancer showed contradictory results (Table 1). Poschke *et al.*^31^ reported increased levels of Glu, Ser, Gln, Ala, Val, Phe, Ile and Leu in 41 breast cancer patients. One possible explanation could be that the stage of tumor in this study population was categorized as early stage such that it did not reduce the amino acid pool. The increased level of Ser was probably due to the increased enzymatic activity involved in Ser biosynthesis in tumor cells.^47^ The increased levels of Glu and Ala may be produced by tumor cells.^48^ Similar increment of amino acid levels, that is, Orn, Glu and Trp in breast cancer patients, was observed previously.^49^ However, other study demonstrated a decrease of Gln, Tyr, Phe, His and Trp, whereas an increase of Thr, Ser, Pro, Gly, Ala, Orn and Lys, in 196 patients with breast cancer.^29^ The above mentioned contradictory results might be attributed to the differences in participant characteristics, including age, gender, ethnic background, diet, and countries where participants live, different measurement techniques applied for PFAA profiles, different diseases stage, and lack of data adjustment for potential confounders. Meanwhile, PFAA profiles may be affected by various factors, including the amount and/or composition of dietary protein, metabolism of muscle protein, as well as the labile protein reserve in different tissues.

## Mechanism underlying alterations of PFAA profiles

Table 1 shows that patients with cancers have altered PFAA profiles. Apparently, the pattern and degree of the alterations depend on the type of cancer and the disease stage. Determination of the precise mechanism underlying changes in the PFAA profiles has the great potential for cancer diagnosis and treatment. Various recent studies tried to find out the connections between cancers and specific PFAA profiles (Table 2). Among all of the amino acids, Gln has attracted great attentions as cancer cells are known to be avid consumers of Gln.^50, 51^ Building on the Warburg effect,^52^ cancer cells extensively use Gln to produce ATP (adenosine-triphosphate) to sustain anabolism, which is necessary for tumor growth and proliferation. It has been demonstrated that breast cancer cell lines expressing high levels of c-*MYC* were dependent on Gln for their survival and growth.^53^ As shown in Table 1, significant decrease of Gln was observed in patients with pancreatic cancer,^27, 38^ Lung cancer, gastric cancer, colorectal cancer, breast cancer and prostate cancer.^29^ On the other hand, the increased Glu levels in colorectal cancer patients^30^ and breast cancer patients^30, 31, 32^ could also be interpreted as the result of increased Gln metabolism in tumor cells. Although Gln consumption is increased in most tumors, some cancer cells can survive and proliferate by relying on glucose without Gln.^54^

Gly and Ser are biosynthetically linked, both of which are classic metabolites of glycolysis. The biosynthesis pathway of Ser utilizes the glycolysis intermediate 3-phosphoglycerate, which is converted by phosphoglycerate dehydrogenase, phosphoserine aminotransferase and phosphoserine phosphatise into Ser. In Gly metabolism, Gly is converted to methylenetetrahydrofolate by glycine decarboxylase. Meanwhile, serine hydroxymethyltransferase converts Ser to Gly reversibly, linking the respective pathways of metabolism. Jain *et al.*^55^ reported that Gly biosynthetic pathway was closely linked to cancer cell proliferation. A significant correlation between Gly consumption and cancer cell proliferation was observed, suggesting that Gly uptake and catabolism was able to promote tumourigenesis and malignancy. Additionally, emerging evidence suggested that aberrant activation of the biosynthetic pathway of Ser was an essential process in cancer pathogenesis.^56^ According to previous studies, glycine decarboxylase is highly expressed in several human cancers, including ovarian cancer,^55^ non-small-cell lung carcinoma^57^ and breast cancer.^58^ Phosphoglycerate dehydrogenase (the key enzyme for Ser biosynthesis) expression is normally upregulated in breast cancer and melanoma.^59^ As summarized in Table 1, Gly consumption was pronounced in pancreatic cancer,^27^ breast cancer,^28, 29^ colorectal cancer^41^ and cervical cancer.^42^ While Ser levels were high in some patients with pancreatic cancer,^20^ breast cancer,^31, 32^ lung cancer, and colorectal cancer,^29^ probably due to the overexpression of phosphoglycerate dehydrogenase, other cancers, such as colorectal cancer,^21, 27, 42^ pancreatic cancer,^27^ gastric cancer^28^ and cervical cancer^43^ consumed Ser. Although Ser and Gly could be inter-converted and either of them might be used for one-carbon metabolism and nucleotide synthesis, convincing evidence suggested that cancer cell proliferation were supported by Ser instead of Gly consumption. Gly was believed to be a consequence of the rapid cell proliferation.^60^

It is well-known that the BCAAs have important roles in the maintenance of lean body mass and regulation of skeletal muscle protein metabolism. Therefore, the investigation of BCAAs and their metabolites in the cancer-bearing state, where muscle wasting is a significant comorbidity, is of importance. Elevated plasma BCAAs levels have been observed to raise the risk of future diagnosis of pancreatic cancer by two-fold.^61^ The high BCAA concentrations were attributed to the enhanced whole-body protein breakdown in development of pancreatic ductal adenocarcinoma. Therefore, BCAA profiles could be used as a general marker to diagnose pancreatic ductal adenocarcinoma. In addition, the direct effects of BCAAs on cultured human hepatocellular carcinoma cells have also been reported previously.^62^ It was found that increased BCAAs levels suppressed the hepatocellular carcinoma cell lines proliferation. One of the plausible mechanisms underlying suppression of cancer cells by BCAAs was associated with their capability of inhibition of insulin signals through suppressing the expression of IGF.^63^ It is believed that insulin induced cell proliferation by activating the mitogen-activated protein kinase pathway.^64^ BCAAs have also been reported to accelerate mRNA degradation of insulin-induced vascular endothelial growth factor at the post transcriptional level, downregulating vascular endothelial growth factor expression during the hepatocellular carcinomas development.^65^ Furthermore, BCAAs were reported to induce the apoptosis of liver cancer cell lines by inhibiting insulin-induced phosphatidylinositol-3-kinase (PI3K)/Akt and the nuclear factor-kappa beta (NF-κB) signaling pathways through the mammalian target of rapamycin complex 1- (mTORC1) and complex 2- (mTORC2) dependent mechanisms.^66^

Some other amino acids, such as Tau, have been reported to decrease human cervical cancer cell proliferation in a dose- and time-dependent manner.^67^ It was also suggested that assessment of serum Tau levels in patients with high breast cancer risk was useful in the early diagnosis of malignant changes in breast.^68^ Additionally, plasma Arg levels were lower in patients with cancers, indicating that Arg metabolism may be disturbed in the presence of a malignancy.^27, 44, 69^ Although the metabolic changes of different cancers can determine their own unique PFAA profiles, the role of cancer-specific amino acids remains to be elucidated. Further studies are required to verify the significance of PFAA alterations in cancers development and management.

## Altered PFAA profiles in diabetes mellitus

T2D is characterized by insulin resistance and/or impaired insulin secretion from beta cells. The prevalence of T2D is markedly increasing around the world and the rates of increase show no signs of slowing.^70^ A complete understanding of the pathophysiology of insulin resistance and T2D, or the identification of early stage metabolic alterations, is promising in the study of etiological pathways and may hold the potential to develop preventive strategies. A number of biomarkers, including fasting plasma glucose and glycated hemoglobinA1c, have been proposed as indicators for the estimation of T2D risk.^71^ Yet many populations from overweight to moderately obese have completely normal fasting plasma glucose and hemoglobinA1c, leaving them undiagnosed as pre-diabetics in spite of underlying dysfunctional metabolism. This highlights the fact that only considering glucose metabolism was not sufficient when determining the etiology and consequences of T2D.^72^

More and more metabolomics-based studies have consistently reported the perturbation of normal amino acid metabolism in insulin resistance and T2D in recent years.^73^ Multiple amino acids, particularly BCAAs, have been shown to be modulators of insulin secretion.^74, 75^ Increasing evidence suggests that elevated plasma BCAAs levels may have adverse effects on the regulation of glucose homeostasis, because the oxidation of BCAAs spares glucose utilization in skeletal muscle.^76^ On the other hand, for individuals without significant abnormalities in glucose homeostasis, elevations in BCAAs levels, along with AAAs are also significantly associated with an increased future likelihood of developing T2D^77^ and cardiovascular diseases.^78^ One of the possible mechanisms by which hyperaminoacidemia could promote DM is via hyperinsulinemia leading to pancreatic beta cell exhaustion. The association between insulin resistance and increased circulating BCAAs levels were supported by several other studies with different ethnic groups and degree of obesity.^12, 13, 14, 16, 79, 80^ Table 3 summarizes 16 recent studies reporting the associations between PFAA profiles and insulin resistance and T2D.

Shah *et al.*^13^ conducted a large randomized trial to understand health benefits occurring as a result of weight loss by using high-throughput metabolomic profiling. They found that BCAAs were unique to predict the improvement in HOMA-IR (homeostasis model assessment of insulin resistance), and suggested a potential mechanism for the heterogeneity in health benefits obtained from weight loss. The associations between BCAAs concentrations and adverse metabolic profiles were also observed in children and adolescents. It is noteworthy that the elevations in BCAAs may independently predict future insulin resistance in these participants.^14^ In the Framingham Offspring Study, BCAAs and AAAs were found to have significant relationships with future development of DM.^77^ The combination of three amino acids, that is, Ile, Phe and Tyr, predicted future DM with a four- to six-fold increased risk for participants in top quartile. The combination of Ile, Phe and Tyr also helped to predict future cardiovascular diseases during a long-term follow-up, probably through increased tendency towards the development of atherosclerosis.^78^ The plausible etiology of elevated BCAAs levels in obesity is through the suppression of BCAA catabolism by insulin resistance.^11^ It should be noted that, besides obesity-associated insulin resistance, BCAAs levels were also positively correlated with HOMA-IR in individuals with normal body mass.^16, 80^ The underlying mechanisms between insulin resistance and elevated BCAAs are related with the persistent activations of mTORC and S6K1 as shown in Table 4.

Increased circulating levels of Phe and Tyr (or AAAs) have been reported to be associated with insulin resistant, T2D or cardiovascular diseases states.^77, 78, 79, 80, 82, 84, 87, 88^ The directionality of the blood concentration shifts of Phe and Tyr are usually the same because Tyr is the first product of Phe catabolism. In the studies using blood metabolites to predict T2D^77^ and determining correlations between metabolites and insulin sensitivity,^89^ Phe and Tyr provided some of the strongest associations. The positive correlation between Phe and/or Tyr and insulin secretion may be involved in pathways to compensate early stage of insulin resistance through stimulating insulin secretion (Table 4).

Contrary to BCAAs and AAAs, the relationships of other amino acids with insulin resistance remain incompletely understood. Nakamura *et al.*^81^ recruited 51 of patients with T2D and measured their PFAA profiles. They observed that the levels of Glu, Ala, Trp and BCAAs were inversely correlated with adiponectin concentrations. As adiponectin is very important in the regulation of insulin sensitivity and metabolism, it might be the cause of insulin resistance and change PFAA profiles in diabetic patients. They also found that the concentrations of Ala, Tyr, Glu and Pro were significantly correlated with fasting plasma insulin. There results indicated the strong association between PFAA profiles, circulating adiponectin concentration and insulin resistance; however, the underlying mechanism was unclear. Some other studies in healthy obese^82, 83^ and in pre-diabetic subjects^84^ suggested that the levels of Ala, Gly, Gln, Glu, Trp, Tyr and BCAAs were correlated with visceral adiposity which was associated with deregulated insulin signaling. However, in the EPIC-Potsdam case–cohort,^85^ increased concentration of Phe and reduced concentration of Gly were found to be independently predictive of T2D. Unlike Phe which stimulated insulin secretion, the depletion of Gly may reflect increased gluconeogenesis or glutathione consumption driven by oxidative stress.^90^

## Conclusions and future directions

**Figure 1:**
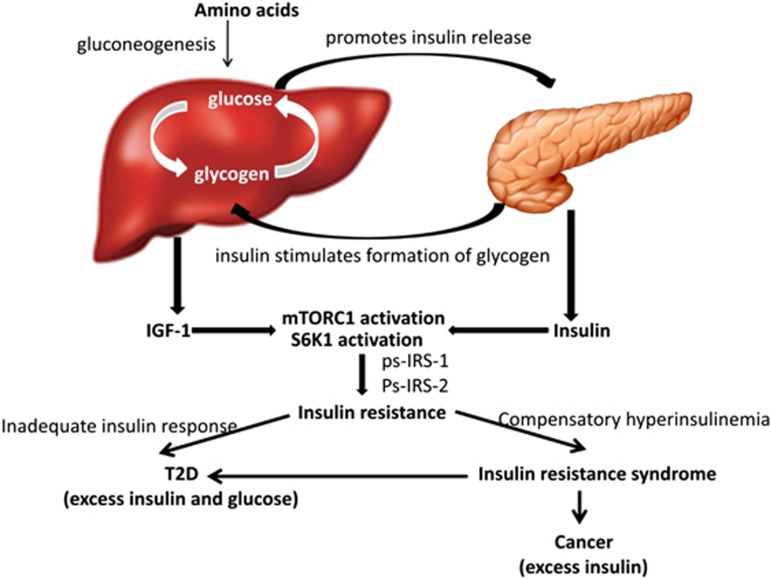
Mechanism of the link between insulin resistance, T2D and cancers. Amino acids metabolism in the liver via the gluconeogenic pathway increases glucose levels, which promotes insulin secretion from the pancreas. IGF-1 and insulin activate mTORC1 and S6K1. Persistent activation leads to serine phosphorylation of IRS-1 and IRS-2. The resulting insulin resistance increases demand on insulin to dispose of excess glucose. Long-term demand may negatively affect the function of islets, resulting in an inadequate insulin response and leading to the onset of T2D. On the other hand, some insulin resistant individuals who are able to maintain the degree of hyperinsulinemia needed to maintain normal (or near-normal) glucose tolerance are at increased risk of cancers. IGF-1, Insulin-like growth factor 1; IRS, insulin receptor substrate; mTORC1, mammalian target of rapamycin complex 1; S6K1, ribosomal protein S6 kinase β1; T2D, type 2 diabetes.

This review has highlighted the potential use of the PFAA profiling as a novel diagnostic tool to access the risk of cancers and T2D. Results from epidemiological studies have suggested that obesity and T2D are positively correlated with the increased risk of several cancers. The underlying link between obesity, T2D, and cancer is related to insulin resistance, hyperinsulinemia, and disturbances in IGF signaling systems (Figure 1). The insulin resistant state is correlated with a metabolic profile of altered metabolism of protein, which may affect the PFAA profiles. Looking at previous clinical data, the metabolic alterations of insulin resistance, T2D or cancers can determine their own unique PFAA profiles. Although PFAA alterations can be used for diagnosis of cancers or T2D with sufficient sensitivity and robustness, the specificity is low. The discrepancies exist between the results of previous studies due to the variations in participant characteristics, for example, age, gender, ethnic background, degree of obesity, diet, different techniques for amino acids measurement, different types and stages of cancers and limited size of data set.

Future research is needed to investigate the characteristic PFAA profiles to discriminate individual cancer types with different stages from healthy controls. Additional validation of the profiles using a larger sample size is necessary to establish the clinical utility. Furthermore, it is needed to elucidate the biological mechanisms by which amino acids might promote cancer risk and progression or T2D and its complications because the roles of insulin resistance or hyperinsulinemia or hyperglycemia in regulating the enzymes utilizing amino acids are still incompletely understood. Although our understanding of alterations in PFAAs metabolism in the diabetic or cancer states remains immature, we believe that PFAA profiling has the clinical usefulness for the detection of cancers or T2D.

On the other hand, while it is evident that Asian populations are more insulin resistant than other ethnic groups, in spite of less obesity, it is necessary to better identify the factors underlying the interethnic differences. As mentioned earlier, PFAA profiles have been utilized as biomarkers to detect cancers and diabetes. However, few studies have been investigated whether specific populations in Asia may have different PFAA profiles. To our best knowledge, only a few studies have been conducted in Asian populations, most of which are limited to Japanese. The rising prevalence of diabetes and cancers in Asia urgently need to clarify the associations of PFAA profiles with these diseases. These findings may provide new insight into how dietary or other interventions alter PFAA profiling in humans and to access whether these changes could ultimately improve metabolic health in cancer patients or pre-diabetes or T2D patients.

## Figures and Tables

**Table 1:** Summary of 20 studies on PFAA profiles in cancer patients (from 2005 to 2015)

*First author*	*Year*	*Disease groups*	n	*PFAA profiles*	*Main conclusions*
Vissers *et al.*^27^	2005	Breast cancer Colonic cancer Pancreatic cancer	22 9 21	Val, Phe, Trp, Leu, Arg, BCAAs↓ Ser, Tau, Tyr, Val, Phe, Trp, Leu, Arg, BCAAs↓ Asn, Ser, Gln, Gly, Thr, His, Cit, Ala, Tau, aAba, Tyr, Val, Met, Ile, Phe, Trp, Leu, Lys, Orn, BCAAs, TAAs↓	Arg decreased in cancer patients both with and without weight loss, irrespective of tumor type and stage.
Gu *et al.*^28^	2015	Gastric cancer Breast cancer Thyroid cancer	56 28 33	Thr, Cys, Arg, EAAs↑ Asp, Ser, Glu, Gly, Ala, Val, Lys, His, Pro, NEAAs, BCAAs, TAAs↓ Thr, Arg, EAAs↑ Asp, Glu, Gly, His, Pro, NEAAs↓ Thr, Met, Leu, Tyr, Lys, Arg, EAAs↑ Asp, Glu, Gly, Pro, NEAAs↓	PFAA patterns in cancer patients are altered perioperatively.
Miyagi *et al.*^29^	2011	Lung cancer Gastric cancer Colorectal cancer Breast cancer Prostate cancer	200 199 199 196 134	Ser, Pro, Gly, Ile, Leu, Phe, Orn↑ Asn, Gln, Cit, His, Trp↓ Thr, Asn, Gln, Ala, Cit, Val, Met, Ile, Leu, Tyr, Phe, His, Trp, Lys, Arg↓ Thr, Asn, Gln, Cit, Val, Met, Leu, Tyr, Phe, His, Trp, Orn, Lys, Arg↓ Thr, Ser, Pro, Gly, Ala, Orn, Lys↑ Gln, Tyr, Phe, His, Trp↓ Thr, Ser, Pro, Gly, Ala, Orn, Lys↑ Gln, Val, Leu, Trp, Arg↓	PFAA profiling had great potential for cancer screening and diagnosis.
Okamoto *et al.*^30^	2009	Colorectal cancer Breast cancer	49 45	Glu↑ Thr, Cit, Val, Met, Ile, Leu, Tyr, Phe, EAAs, BCAAs↓ Thr, Ser, Glu, aAba, Orn↑ Met, Ile, Phe, Arg↓	The development of a cancer alters PFAA profiles without cachexia or weight loss.
Poschke *et al.*^31^	2013	Breast cancer	41	Glu, Ser, Gln, Ala, Val, Phe, Ile, Leu↑	Increased amino acid levels correlated with pro-inflammatory state and intrinsic tumor subtype.
Barnes *et al.*^32^	2014	Breast cancer	8	Glu, His↑ Thr, Tau, Leu↓	The altered PFAA profiles may be related to metabolic perturbations associated with breast cancer.
Nagata *et al.*^33^	2014	Breast cancer	350 normal premenopausal Japanese women Plasma levels of Arg, Leu, Tyr, and Asn were associated with the levels of sex hormones and IGF-1, which were relevant to the etiology of premenopausal breast cancer.		
Shingyogi *et al.*^34^	2013	Lung cancer	171	Pro, Orn↑ His↓	PFAA profiling has great potential for early detection of lung cancer.
Zhao *et al.*^35^	2014	Lung cancer	27	Phe, Asp, Hyl↑ Trp, Gly, Cit, Orn, Pro↓	PFAAs can be used to detect lung cancer patients due to their stronger correlation with cancer tissue free amino acids.
Kim *et al.*^36^	2015	Lung cancer	72	Pro, Ile, Phe, Orn↑ Thr, Cit, His, Trp↓	In the early stages (I and II), significant decreases in the relative levels of Thr, Cit, His and Trp and increases in Pro and Ile. Significant increases in Phe and Orn during the late stages (III and IV).
Fukutake *et al.*^20^	2015	Pancreatic cancer	360	Ser↑ Thr, Asn, Pro, Ala, Cit, Val, Met, Leu, Tyr, Phe, His, Trp, Lys, Arg↓	PFAA index was developed and validated for detection of pancreatic cancer.
Schrader *et al.*^37^	2009	Pancreatic cancer	12	Thr, Val, Trp, Lys, Asp, Ala, Arg, Tau, Cit, EAAs, NEAAs, TAAs↓	A significant deficit in circulating amino acid levels in pancreatic cancer patients.
Kobayashi *et al.*^38^	2013	Pancreatic cancer	43	Val, Thr, Met, Asn, Gln, Lys, His, Tyr↓	Amino acid profiles and other metabolomics can be used as a diagnostic method for pancreatic cancer.
Ma *et al.*^39^	2014	Esophageal cancer	51	Asp, Cys↑ Glu, Gly, His, Thr, Tau, Ala, Met, Ile, Leu, Phe↓	PFAA profiles in esophageal cancer patients are significantly different from those in controls.
Zhang *et al.*^40^	2012	Esophageal cancer	67	Gln, Lys↑ Val, Leu/Ile, Met, Tyr, Trp↓	Metabolic profiles can distinguish esophageal cancer patients.
Yatabe *et al.*^21^	2013	Colorectal cancer	Case report	High plasma level of Ile and low level of Ser	An early stage of colon carcinoma is found from PFAA distribution.
Leichtle *et al.*^41^	2012	Colorectal cancer	59	Lys, Ala, Asp, Gly, His, Leu/Ile, Met, Sarc, Thr, Tyr, Val↓	The significant alterations in the amino acid profile in colon cancer have promising diagnostic power.
Hasim *et al.*^42^	2013	Cervical cancer	22	Asp, Glu, Asn, Ser, Gly, His, Arg, Thr, Tau, Tyr, Val, Met, Lys, Ile, Leu, Phe↓	PFAA profiles may have the potential for cancer diagnoses in the early stage.
Ihata *et al.*^43^	2014	Endometrial cancer	80	Orn, Ile, Pro↑ His, Trp, Val, Phe, Asn, Ser, Leu, Met↓	Amino acid profile index has a significant role in the preoperative evaluation of endometrial cancer.
Buijs *et al.*^44^	2010	Head and neck cancer	32	Arg may be a potential player in the treatment of head and neck cancer patients: perioperative Arg-enriched enteral nutrition improved the long-term overall and disease-specific survival in malnourished patients with head and neck cancer	

Abbreviations: BCAA, branched-chain amino acid; IGF-1, insulin-like growth factor 1; PFAA, plasma-free amino acid; TAA, total amino acid.

**Table 2:** Connections between amino acids and different cancers

*Amino acids*	*Cancer type*	*Mechanisms*
Gln↓	Breast cancer^29^ Pancreatic cancer^27, 38^ Lung cancer^29^ Gastric cancer^29^ Colorectal cancer^29^ Prostate cancer^29^	Cancer cells depend on Gln for tricarboxylic acid cycle intermediates, cellular energetics, reactive oxygen species buffering, lipid synthesis, mTOR activity, and autophagy (which is inhibited by mTOR).
Gly↓	Breast cancer^28, 29^ Pancreatic cancer^27^ Colorectal cancer^41^ Cervical cancer^42^	Gly consumption and its mitochondrial biosynthetic pathway are strongly correlated with cancer cell proliferation.
Ser↓	Pancreatic cancer^27^ Gastric cancer^28^ Colorectal cancer^21, 27, 42^ Cervical cancer^43^	Cancer cells consumed Ser to convert to intracellular Gly and one-carbon units for building nucleotides.
BCAAs↓	Breast cancer^27^ Pancreatic cancer^27^ Gastric cancer^28^ Colorectal cancer^30^ Colonic cancer^27^	The muscle wasting syndrome experience by many cancer patients decreased BCAAs levels.

Abbreviations: BCAA, branched-chain amino acid; mTOR, mammalian target of rapamycin.

**Table 3:** Associations between PFAAs and insulin resistance and T2D (from 2010 to 2016)

*First author*	*Year*	*Subjects*	n	*Main conclusions*
Shah *et al.*^13^	2012	Non-diabetic individuals	500 (62.6% White and 37.4% African-American)	BCAA was correlated with insulin resistance and predicted improvement in HOMA-IR, independent of the amount of weight lost.
McCormack *et al.*^14^	2013	Healthy subjects, ages 8–18 years	69 (29 girls and 40 boys; Among them, 21 African-American, 36 White and 12 others)	Elevated circulating BCAAs levels were significantly associated with obesity in children and adolescents, and may predict future insulin resistance independently.
Wurtz *et al.*^15^	2013	Finnish cohorts	1680 (769 men and 911 women)	BCAAs and AAAs are markers of the development of insulin resistance in young, normoglycemic adults.
Tai *et al.*^16^	2010	Chinese and Asian-Indian	263 men (180 Chinese and 83 Asian-Indian)	Ala, Tyr, and Gln/Glu were positively correlated with HOMA-IR for both ethnic groups. Pro, Val, Leu/Ile, and Orn were positively correlated with HOMA-IR among Chinese participants, but not Asian-Indian participants.
Wang *et al.*^77^	2011	Framingham Offspring Study	778 (388 men and 390 women)	BCAAs and AAAs had significant associations with future development of diabetes.
Magnusson *et al.*^78^	2013	Malmö Diet and Cancer Study	1297 (647 men and 650 women)	The combination of Ile, Tyr, and Phe predicted cardiovascular disease events during long-term follow-up.
Wurtz *et al.*^79^	2012	Finnish cohorts	7098 (3433 men and 3665 women)	Circulating amino acids displayed sex- and obesity-dependent interactions with HOMA-IR. The associations of HOMA-IR with Ile, Leu, Val, Phe, Tyr and Ala were significant for all men, whereas only obese women displayed associations for Ile, Leu, Val, and Tyr. Gln was inversely associated with HOMA-IR for obese men, but the association was present for all women.
Yamada *et al.*^80^	2015	Non-diabetic Japanese	94 (48 men and 46 women)	Ile, Leu, Phe, Tyr and BCAAs were significantly positively correlated with HOMA-IR.
Nakamura *et al.*^81^	2014	T2D patients	51 (23 men and 28 women)	Ala, Tyr, Glu and Pro were strongly associated with hyperinsulinemia, while Ala, Glu, Trp and BCAAs were associated with hypoadiponectinemia.
Martin *et al.*^82^	2013	Healthy women	40	Plasma Gln, Leu/Ile, Phe and Tyr significantly contributed to visceral adiposity.
Yamakado *et al.*^83^	2012	Obese Japanese subjects	1449 (985 men and 464 women)	Accumulated visceral fat altered the peripheral amino acid profile; A multivariate logistic regression model of PFAAs could distinguish visceral obesity.
Wang-Sattler *et al.*^84^	2012	Cooperative Health Research in the Region of Augsburg (KORA) cohort	1297 (cross-sectional study) 1465 (prospective study)	The decreased Gly concentrations in individuals with impaired glucose tolerance and T2D may result from insulin resistance. The change might precede other BCAAs and AAAs in the progression of T2D.
Floegel *et al.*^85^	2013	EPIC-Potsdam case–cohort	2282 subcohort (62.0% women) and 800 incident T2D (42.2% women)	Higher concentration of Phe was associated with increased risk of T2D and Gly was associated with reduced risk of T2D.
Kume *et al.*^86^	2014	Japanese patients with T2D	385 (63 developed cardiovascular composite endpoints)	aABA, 3MeHis and Cit were significantly higher and Trp was significantly lower in patients developed cardiovascular composite endpoints.
Yamakado *et al.*^87^	2015	Japanese	2984 (1877 men)	Ile, Leu, Tyr and Phe were significantly related to the development of DM; Ile, Leu, Tyr, Ala and Ser were significantly related to the development of metabolic syndrome; Ile, Leu, Tyr, Val, Ala, Pro, Ser and Gly significantly related to the development of dyslipidemia.
Wurtz *et al.*^88^	2012	Finnish cohorts	1873 (58% women)	Alterations in BCAAs and AAAs metabolism precede hyperglycemia in the general population.

Abbreviations: AAA, aromatic amino acid; BCAA, branched-chain amino acid; HOMA-IR, homeostasis model assessment of insulin resistance; PFAA, plasma-free amino acid; T2D, type 2 diabetes.

**Table 4:** Connections between amino acids and insulin resistance

*Amino acids*	*Mechanisms*
BCAAs	The elevated BCAAs are able to activate mTORC and its downstream effecter S6K1 in the liver, muscle and adipose tissue. Persistent activation leads to serine phosphorylation of IRS-1 and thus the inhibition of IRS-1, resulting in insulin resistance.
AAAs	AAAs are metabolized to catecholamines, which alter the liver function leading to hyperinsulinemia and dyslipidemia.
Met	Met metabolism may intersect with Phe/Tyr catabolism and stimulate insulin secretion. Its transmethylation to homocysteine to affect glucose homeostasis.
Ala	Ala is metabolized to pyruvate to maintain glucose homeostasis.

Abbreviations: AAA, aromatic amino acid; BCAA, branched-chain amino acid; mTORC, mammalian target of rapamycin complex.

## References

[bib1] Vigneri P, Frasca F, Sciacca L, Pandini G, Vigneri R. Diabetes and cancer. Endocr Relat Cancer 2009; 16: 1103–1123. 19620249 10.1677/ERC-09-0087

[bib2] Giovannucci E, Harlan DM, Archer MC, Bergenstal RM, Gapstur SM, Habel LA et al. Diabetes and cancer: a consensus report. Diabetes Care 2010; 33: 1674–1685. 20587728 10.2337/dc10-0666 PMC2890380

[bib3] Joh HK, Willett WC, Cho E. Type 2 diabetes and the risk of renal cell cancer in women. Diabetes Care 2011; 34: 1552–1556. 21602426 10.2337/dc11-0132 PMC3120193

[bib4] Kahn SE, Hull RL, Utzschneider KM. Mechanism linking obesity to insulin resistance and type 2 diabetes. Nature 2006; 444: 840–846. 17167471 10.1038/nature05482

[bib5] Solarek W, Czarnecka AM, Escudier B, Bielecka ZF, Lian F, Szczylik C. Insulin and IGFs in renal cancer risk and progression. Endocr Relat Cancer 2015; 22: R253–R264. 26330483 10.1530/ERC-15-0135

[bib6] Savage DB, Petersen KF, Shulman GI. Disordered lipid metabolism and the pathogenesis of insulin resistance. Physiol Rev 2007; 87: 507–520. 17429039 10.1152/physrev.00024.2006 PMC2995548

[bib7] Muoio DM, Newgard CB. Mechanisms of diseases: molecular and metabolic mechanisms of insulin resistance and beta-cell failure in type 2 diabetes. Nat Rev Mol Cell Biol 2008; 9: 193–205. 18200017 10.1038/nrm2327

[bib8] Kim YS, Maruvada P, Milner JA. Metabolomics in biomarker discovery: future uses for cancer prevention. Future Oncol 2008; 4: 93–102. 18241004 10.2217/14796694.4.1.93

[bib9] Felig P, Marliss E, Cahill GFJr. Plasma amino acid levels and insulin secretion in obesity. N Engl J Med 1969; 281: 811–816. 5809519 10.1056/NEJM196910092811503

[bib10] Costeas PA, Chinsky JM. Effects of insulin on the regulation of branched-chain alpha-keto acid dehydrogenase E1 alpha subunit gene expression. Biochem J 1996; 318: 85–92. 8761456 10.1042/bj3180085 PMC1217592

[bib11] Lackey DE, Lynch CJ, Olson KC, Mostaedi R, Ali M, Smith WH et al. Regulation of adipose branched-chain amino acid catabolism enzyme expression and cross-adipose amino acid flux in human obesity. Am J Physiol Endocrinol Metab 2013; 304: E1175–E1187. 23512805 10.1152/ajpendo.00630.2012 PMC3680678

[bib12] Newgard CB, An J, Bain JR, Muehlbauer MJ, Stevens RD, Lien LF et al. A branched-chain amino acid-related metabolic signature that differentiates obese and lean humans and contributes to insulin resistance. Cell Metab 2009; 9: 311–326. 19356713 10.1016/j.cmet.2009.02.002 PMC3640280

[bib13] Shah SH, Crosslin DR, Haynes CS, Nelson S, Turer CB, Stevens RD et al. Branched-chain amino acid levels are associated with improvement in insulin resistance with weight loss. Diabetologia 2012; 55: 321–330. 22065088 10.1007/s00125-011-2356-5 PMC3667157

[bib14] McCormack SE, Shaham O, McCarthy MA, Deik AA, Wang TJ, Gerszten RE et al. Circulating branched-chain amino acid concentrations are associated with obesity and future insulin resistance in children and adolescents. Pediatr Obes 2013; 8: 52–61. 22961720 10.1111/j.2047-6310.2012.00087.x PMC3519972

[bib15] Wurtz P, Soininen P, Kangas AJ, Rönnemaa T, Lehtimäki T, Kähönen M et al. Branched-chain and aromatic amino acids are predictors of insulin resistance in young adults. Diabetes Care 2013; 36: 648–655. 23129134 10.2337/dc12-0895 PMC3579331

[bib16] Tai ES, Tan MLS, Stevens RD, Low YL, Muehlbauer MJ, Goh DLM et al. Insulin resistance is associated with a metabolic profile of altered protein metabolism in Chinese and Asian-Indian men. Diabetologia 2010; 53: 757–767. 20076942 10.1007/s00125-009-1637-8 PMC3753085

[bib17] Wang TJ, Larson MG, Vasan RS, Cheng S, Rhee EP, McCabe E et al. Metabolite profiles and the risk of developing diabetes. Nat Med 2011; 17: 448–453. 21423183 10.1038/nm.2307 PMC3126616

[bib18] Engelen MP, Wouters EF, Deutz NE, Menheere PP, Schols AM. Factors contributing to alterations in skeletal muscle and plasma amino acid profiles in patients with chronic obstructive pulmonary disease. Am J Clin Nutr 2000; 72: 1480–1487. 11101475 10.1093/ajcn/72.6.1480

[bib19] Zhang Q, Takahashi M, Noguchi Y, Sugimoto T, Kimura T, Okumura A et al. Plasma amino acid profiles applied for diagnosis of advanced liver fibrosis in patients with chronic hepatitis C infection. Hepatol Res 2006; 34: 170–177. 16436332 10.1016/j.hepres.2005.12.006

[bib20] Fukutake N, Ueno M, Hiraoka N, Shimada K, Shiraishi K, Saruki N et al. A novel multivariate index for pancreatic cancer detection based on the plasma free amino acid profile. PLoS One 2015; 10: e0132223. 26133769 10.1371/journal.pone.0132223 PMC4489861

[bib21] Yatabe J, Yatabe MS, Ishibashi K, Nozawa Y, Sanada H. Early detection of colon cancer by amino acid profiling using AminoIndex Technology: a case report. Diagn Pathol 2013; 8: 203. 24325735 10.1186/1746-1596-8-203 PMC3937238

[bib22] Noguchi Y, Zhang QW, Sugimoto T, Furuhata Y, Sakai R, Mori M et al. Network analysis of plasma and tissue amino acids and the generation of an amino index for potential diagnostic use. Am J Clin Nutr 2006; 83: 513S–519S. 16470023 10.1093/ajcn/83.2.513S

[bib23] Huang J, Plass C, Gerheauser C. Cancer chemoprevention by targeting the epigenome. Curr Drug Targets 2011; 12: 1925–1956. 21158707 10.2174/138945011798184155

[bib24] Stattin P, Bylund A, Rinaldi S, Biessy C, Dechaud H, Stenman UH et al. Plasma insulin-like growth factor-1, insulin-like growth factor-binding proteins, and prostate cancer risk: a prospective study. J Natl Cancer Inst 2000; 92: 1910–1917. 11106682 10.1093/jnci/92.23.1910

[bib25] Burroughs KD, Dunn SE, Barrett JC, Taylor JA. Insulin-like growth factor-1: a key regulator of human cancer risk. J Natl Cancer Inst 1999; 91: 579–581. 10203270 10.1093/jnci/91.7.579

[bib26] Proenza AM, Oliver J, Palou A, Roca P. Breast and lung cancer are associated with a decrease in blood cell amino acid content. J Nutr Biochem 2003; 14: 133–138. 12742540 10.1016/s0955-2863(02)00225-5

[bib27] Vissers YL, Dejong CH, Luiking YC, Fearon KC, Meyenfeldt MF, Deutz NE. Plasma arginine concentrations are reduced in cancer patients: evidence for arginine deficiency? Am J Clin Nutr 2005; 81: 1142–1146. 15883440 10.1093/ajcn/81.5.1142

[bib28] Gu Y, Chen T, Fu S, Sun X, Wang L, Wang J et al. Perioperative dynamics and significance of amino acid profiles in patients with cancer. J Transl Med 2015; 13: 35. 25622826 10.1186/s12967-015-0408-1 PMC4332895

[bib29] Miyagi Y, Higashiyama M, Gochi A, Akaike M, Ishikawa T, Miura T et al. Plasma free amino acid profiling of five types of cancer patients and its application for early detection. PLoS One 2011; 6: e24143. 21915291 10.1371/journal.pone.0024143 PMC3168486

[bib30] Okamoto N, Miyagi Y, Chiba A, Akaike M, Shiozawa M, Imaizumi A et al. Diagnostic modeling with differences in plasma amino acid profiles between non-cachectic colorectal/breast cancer patients and healthy individuals. Int J Med Med Sci 2009; 1: 1–8.

[bib31] Poschke I, Mao Y, Kiessling R, Boniface J. Tumor-dependent increase of serum amino acid levels in breast cancer patients has diagnostic potential and correlates with molecular tumor subtypes. J Transl Med 2013; 11: 290. 24237611 10.1186/1479-5876-11-290 PMC3835137

[bib32] Barnes T, Bell K, DiSebastiano KM, Vance V, Hanning R, Russell C et al. Plasma amino acid profiles of breast cancer patients early in the trajectory of the disease differ from healthy comparison groups. Appl Physiol Nutr Metab 2014; 39: 740–744. 24819038 10.1139/apnm-2013-0526

[bib33] Nagata C, Wada K, Tsuji M, Hayashi M, Takeda N, Yasuda K. Plasma amino acid profiles are associated with biomarkers of breast cancer risk in premenopausal Japanese women. Cancer Causes Control 2014; 25: 143–149. 24186145 10.1007/s10552-013-0316-8

[bib34] Shingyoji M, Iizasa T, Higashiyama M, Imamura F, Saruki N, Imaizumi A et al. The significance and robustness of a plasma free amino acid (PFAA) profile-based multiplex function for detecting lung cancer. BMC Cancer 2013; 13: 77. 23409863 10.1186/1471-2407-13-77 PMC3598471

[bib35] Zhao Q, Cao Y, Wang Y, Hu C, Hu A, Ruan L et al. Plasma and tissue free amino acid profiles and their concentration correlation in patients with lung cancer. Asia Pac J Clin Nutr 2014; 23: 429–436. 25164454 10.6133/apjcn.2014.23.3.13

[bib36] Kim HJ, Jang SH, Ryu JS, Lee JE, Kim YC, Lee MK et al. The performance of a novel amino acid multivariate index for detecting lung cancer: a case control study in Korea. Lung Cancer 2015; 90: 522–527. 26476713 10.1016/j.lungcan.2015.10.006

[bib37] Schrader H, Menge BA, Belyaev O, Uhl W, Schmidt WE, Meier JJ. Amino acid malnutrition in patients with chronic pancreatitis and pancreatic carcinoma. Pancreas 2009; 38: 416–421. 19169171 10.1097/MPA.0b013e318194fc7a

[bib38] Kobayashi T, Nishiumi S, Ikeda A, Yoshie T, Sakai A, Matsubara A et al. A novel serum metabolomics-based diagnostic approach to pancreatic cancer. Cancer Epidemiol Biomarkers Prev 2013; 22: 571–579. 23542803 10.1158/1055-9965.EPI-12-1033

[bib39] Ma H, Hasim A, Mamtimin B, Kong B, Zhang HP, Sheyhidin I. Plasma free amino acid profiling of esophageal cancer using high-performance liquid chromatography spectroscopy. World J Gastroenterol 2014; 20: 8653–8659. 25024622 10.3748/wjg.v20.i26.8653 PMC4093717

[bib40] Zhang J, Bowers J, Liu L, Wei S, Gowda GA, Hammoud Z et al. Esophageal cancer metabolite biomarkers detected by LC-MS and NMR methods. PLoS One 2012; 7: e30181. 22291914 10.1371/journal.pone.0030181 PMC3264576

[bib41] Leichtle AB, Nuoffer JM, Ceglarek U, Kase J, Conrad T, Witzigmann H et al. Serum amino acid profiles and their alterations in colorectal cancer. Metabolomics 2012; 8: 643–653. 22833708 10.1007/s11306-011-0357-5 PMC3397217

[bib42] Hasim A, Aili A, Maimaiti A, Mamtimin B, Abudula A, Upur H. Plasma-free amino acid profiling of cervical cancer and cervical intraepithelial neoplasia patients and its application for early detection. Mol Biol Rep 2013; 40: 5853–5859. 24068431 10.1007/s11033-013-2691-3

[bib43] Ihata Y, Miyagi E, Numazaki R, Muramatsu T, Imaizumi A, Yamamoto H et al. Amino acid profile index for early detection of endometrial cancer: verification as a novel diagnostic marker. Int J Clin Oncol 2014; 19: 364–372. 23700142 10.1007/s10147-013-0565-2

[bib44] Buijs N, van Bokhorst-de van der Schueren MA, Langius JA, Leemans CR, Kuik DJ, Vermeulen MA et al. Perioperative arginine-supplemented nutrition in malnourished patients with head and neck cancer improves long-term survival. Am J Clin Nutr 2010; 92: 1151–1156. 20881073 10.3945/ajcn.2010.29532

[bib45] Maeda J, Higashiyama M, Imaizumi A, Nakayama T, Yamamoto H, Daimon T et al. Possibility of multivariate function composed of plasma amino acid profiles as a novel screening index for non-small cell lung cancer: a case control study. BMC Cancer 2010; 10: 690. 21176209 10.1186/1471-2407-10-690 PMC3014908

[bib46] Okamoto N. Use of 'AminoIndex Technology' for cancer screening. Ningen Dock 2012; 26: 911–922.

[bib47] Medina MA, Marquez J, Nunez de Castro I. Interchange of amino acids between tumor and host. Biochem Med Metab Biol 1992; 48: 1–7. 1524866 10.1016/0885-4505(92)90041-v

[bib48] Marquez J, Sanchez-Jimenez F, Medina MA, Quesada AR, Nunez de Castro I. Nitrogen metabolism in tumor bearing mice. Arch Biochem Biophys 1989; 268: 667–675. 2913952 10.1016/0003-9861(89)90335-4

[bib49] Cascino A, Muscaritoli M, Cangiano C, Coversano L, Laviano A, Ariemma S et al. Plasma amino acid imbalance in patients with lung and breast cancer. Anticancer Res 1995; 15: 507–510. 7763031

[bib50] Medina MA. Glutamine and cancer. J Nutr 2001; 131: 2539S–2542S. 11533309 10.1093/jn/131.9.2539S

[bib51] Chen L, Cui H. Targeting glutamine induces apoptosis: a cancer therapy approach. Int J Mol Sci 2015; 16: 22830–22855. 26402672 10.3390/ijms160922830 PMC4613338

[bib52] Warburg O, Wind F, Negelein E. The metabolism of tumors in the body. J Gen Physiol 1927; 8: 519–530. 19872213 10.1085/jgp.8.6.519 PMC2140820

[bib53] Korangath P, Teo WW, Sadik H, Han L, Mori N, Huijts CM et al. Targeting glutamine metabolism in breast cancer with aminooxyacetate. Clin Cancer Res 2015; 21: 3263–3273. 25813021 10.1158/1078-0432.CCR-14-1200 PMC4696069

[bib54] Cheng T, Sudderth J, Yang C, Mullen AR, Jin ES, Mates JM et al. Pyruvate carboxylase is required for glutamine-independent growth of tumor cells. Proc Natl Acad Sci USA 2011; 108: 8674–8679. 21555572 10.1073/pnas.1016627108 PMC3102381

[bib55] Jain M, Nilsson R, Sharma S, Madhusudhan N, Kitami T, Souza AL et al. Metabolite profiling identifies a key role for glycine in rapid cancer cell proliferation. Science 2012; 336: 1040–1044. 22628656 10.1126/science.1218595 PMC3526189

[bib56] Amelio I, Cutruzzola F, Antonov A, Agostini M, Melino G. Serine and glycine metabolism in cancer. Trends Biochem Sci 2014; 39: 191–198. 24657017 10.1016/j.tibs.2014.02.004 PMC3989988

[bib57] Zhang WC, Shyh-Chang N, Yang H, Rai A, Umashankar S, Ma S et al. Glycine decarboxylase activity drives non-small cell lung cancer tumor-initiating cells and tumorigenesis. Cell 2012; 148: 259–272. 22225612 10.1016/j.cell.2011.11.050

[bib58] Kim SK, Jung WH, Koo JS. Differential expression of enzymes associated with serine/glycine metabolism in different breast cancer subtypes. PLoS One 2014; 9: 101004. 10.1371/journal.pone.0101004 PMC4076239 24979213

[bib59] Possemato R, Marks KM, Shaul YD, Pacold ME, Kim D, Birsoy K et al. Functional genomics reveal that the serine synthesis pathway is essential in breast cancer. Nature 2011; 476: 346–350. 21760589 10.1038/nature10350 PMC3353325

[bib60] Labuschagne CF, van den Broek NJ, Mackay GM, Vousden KH, Maddocks OD. Serine, but not glycine, supports one-carbon metabolism and proliferation of cancer cells. Cell Rep 2014; 7: 1248–1258. 24813884 10.1016/j.celrep.2014.04.045

[bib61] Mayers JR, Wu C, Clish CB, Kraft P, Torrence ME, Fiske BP et al. Elevation of circulating branched-chain amino acids is an early event in human pancreatic adenocarcinoma development. Nat Med 2014; 20: 1193–1198. 25261994 10.1038/nm.3686 PMC4191991

[bib62] Sugiyama K, Yu L, Nagasue N. Direct effect of branched-chain amino acids on the growth and metabolism of cultured human hepatocellular carcinoma cells. Nutr Cancer 1998; 31: 62–68. 9682250 10.1080/01635589809514679

[bib63] Iwasa J, Shimizu M, Shiraki M, Shirakami Y, Sakai H, Terakura Y et al. Dietary supplementation with branched-chain amino acids suppresses diethylnitrosamine-induced liver tumorigenesis in obese and diabetic C57BL/KsJ-db/db mice. Cancer Sci 2010; 101: 460–467. 19906067 10.1111/j.1349-7006.2009.01402.x PMC11159020

[bib64] Formisano P, Oriente F, Fiory F, Caruso M, Miele C, Maitan MA et al. Insulin-activated protein kinase Cbeta bypasses Ras and stimulates mitogen-activated protein kinase activity and cell proliferation in muscle cells. Mol Cell Biol 2000; 20: 6323–6333. 10938109 10.1128/mcb.20.17.6323-6333.2000 PMC86107

[bib65] Miuma S, Ichikawa T, Arima K, Takeshita S, Muraoka T, Matsuzaki T et al. Branched-chain amino acid deficiency stabilizes insulin-induced vascular endothelial growth factor mRNA in hepatocellular carcinoma cells. J Cell Biochem 2012; 113: 3113–3121. 22581719 10.1002/jcb.24188

[bib66] Hagiwara A, Nishiyama M, Ishizaki S. Branched-chain amino acids prevent insulin-induced hepatic tumor cell proliferation by inducing apoptosis through mTORC1 and mTORC2-dependent mechanisms. J Cell Physiol 2012; 227: 2097–2105. 21769869 10.1002/jcp.22941

[bib67] Kim T, Kim AK. Taurine enhances anticancer activity of cisplatin in human cervical cancer cells. Adv Exp Med Biol 2013; 776: 189–198. 23392883 10.1007/978-1-4614-6093-0_19

[bib68] Agouza EI, Eissa IM, SS, Houseini EI, EI-Nashar MM, Abd DE et al. A novel tumor marker for enhanced detection of breast cancer amnong female patients. Angiogenesis 2011; 14: 321–330. 21553281 10.1007/s10456-011-9215-3

[bib69] Lind DS. Arginine and cancer. J Nutr 2004; 134: 2837S–2841S. 15465796 10.1093/jn/134.10.2837S

[bib70] Wild S, Roglic G, Green A, Sicree R, King H. Global prevalence of diabetes: estimates for the year 2000 and projections for 2030. Diabetes Care 2004; 27: 1047–1053. 15111519 10.2337/diacare.27.5.1047

[bib71] Peters AL, Davidson MB, Schriger DL, Hasselblad V. A clinical approach for the diagnosis of diabetes mellitus: an analysis using glycosylated hemoglobin levels. Meta-analysis research group on the diagnosis of diabetes using glycated hemoglobin levels. JAMA 1996; 276: 1246–1252. 8849753

[bib72] Adams SH. Emerging perspectives on essential amino acid metabolism in obesity and the insulin-resistant state. Adv Nutr 2011; 2: 445–456. 22332087 10.3945/an.111.000737 PMC3226382

[bib73] Fiehn O, Garvey WT, Newman JW, Lok KH, Hoppel CL, Adams SH. Plasma metabolomic profiles reflective of glucose homeostasis in non-diabetic and type 2 diabetic obese African-American women. PLoS ONE 2010; 5: e15234. 21170321 10.1371/journal.pone.0015234 PMC3000813

[bib74] Nilsson M, Holst JJ, Bjorck IM. Metabolic effects of amino acid mixtures and whey protein in healthy subjects: studies using glucose-equivalent drinks. Am J Clin Nutr 2007; 85: 996–1004. 17413098 10.1093/ajcn/85.4.996

[bib75] Van Loon LJ, Saris WH, Verhagen H, Wagenmakers AJ. Plasma insulin responses after ingestion of different amino acid or protein mixtures with carbohydrate. Am J Clin Nutr 2000; 72: 96–105. 10871567 10.1093/ajcn/72.1.96

[bib76] Buse MG, Biggers JF, Friderici KH, Buse JF. Oxidation of branched chain amino acids by isolated hearts and diaphragms of the rat. The effect of fatty acids, glucose, and pyruvate respiration. J Biol Chem 1972; 247: 8085–8096. 4640937

[bib77] Wang TJ, Larson MG, Vasan RS, Cheng S, Phee EP, McCabe E et al. Metabolite profiles and the risk of developing diabetes. Nat Med 2011; 17: 448–454. 21423183 10.1038/nm.2307 PMC3126616

[bib78] Magnusson M, Lewis GD, Ericson U, Orho-Melander M, Hedblad B, Engstrom G et al. A diabetes-predictive amino acid score and future cardiovascular disease. Eur Heart J 2013; 34: 1982–1989. 23242195 10.1093/eurheartj/ehs424 PMC3703309

[bib79] Wurtz P, Makinen VP, Soininen P, Kangas AJ, Tukiainen T, Kettunen J et al. Metabolic signatures of insulin resistance in 7098 young adults. Diabetes 2012; 61: 1372–1380. 22511205 10.2337/db11-1355 PMC3357275

[bib80] Yamada C, Kondo M, Kishimoto N, Shibata T, Nagai Y, Imanishi T et al. Association between insulin resistance and plasma amino acid profile in non-diabetic Japanese subjects. J Diabetes Invest 2015; 6: 408–415. 10.1111/jdi.12323 PMC4511300 26221519

[bib81] Nakamura H, Jinzu H, Nagao K, Noguchi Y, Shimba N, Miyano H et al. Plasma amino acid profiles are associated with insulin, C-peptide and adiponectin levels in type 2 diabetic patients. Nutr Diabetes 2014; 4: e133. 25177913 10.1038/nutd.2014.32 PMC4183973

[bib82] Martin FPJ, Montoliu I, Collino S, Scherer M, Guy P, Tavazzi I et al. Topographical body fat distribution links to amino acid and lipid metabolism in healthy non-obese women. PLoS One 2013; 8: e73445. 24039943 10.1371/journal.pone.0073445 PMC3770640

[bib83] Yamakado M, Tanaka T, Nagao K, Ishizaka Y, Mitushima T, Tani M et al. Plasma amino acid profile is associated with visceral fat accumulation in obese Japanese subjects. Clin Obes 2012; 2: 29–40. 25586045 10.1111/j.1758-8111.2012.00039.x

[bib84] Wang-Sattler R, Yu Z, Herder C, Messias AC, Floegel A, He Y et al. Novel biomarkers for pre-diabetes identified by metabolomics. Mol Syst Biol 2012; 8: 615. 23010998 10.1038/msb.2012.43 PMC3472689

[bib85] Floegel A, Stefan N, Yu Z, Muhlenbruch K, Drogan D, Joost HG et al. Identification of serum metabolites associated with risk of type 2 diabetes using a targeted metabolomic approach. Diabetes 2013; 62: 639–648. 23043162 10.2337/db12-0495 PMC3554384

[bib86] Kume S, Araki S, Ono N, Shinhara A, Muramatsu T, Araki H et al. Predictive properties of plasma amino acid profile for cardiovascular disease in patients with type 2 diabetes. PLoS One 2015; 9: e101219. 10.1371/journal.pone.0101219 PMC4074128 24971671

[bib87] Yamakado M, Nagao K, Imaizumi A, Tani M, Toda A, Tanaka T et al. Plasma free amino acid profiles predict four-year risk of developing diabetes, metabolic syndrome, dyslipidemia, and hypertension in Japanese population. Sci Rep 2015; 5: 11918. 26156880 10.1038/srep11918 PMC4496670

[bib88] Wurtz P, Tiainen M, Makinen VP, Kangas AJ, Soininen P, Saltevo J et al. Circulating metabolite predictors of glycemia in middle-aged men and women. Diabetes Care 2012; 35: 1749–1756. 22563043 10.2337/dc11-1838 PMC3402262

[bib89] Huffman KM, Shah SH, Stevens RD, Bain JR, Muehlbauer M, Slentz CA et al. Relationships between circulating metabolic intermediates and insulin action in overweight to obese, inactive men and women. Diabetes Care 2009; 32: 1678–1683. 19502541 10.2337/dc08-2075 PMC2732163

[bib90] Sekhar RV, McKay SV, Patel SG, Guthikonda AP, Reddy VT, Balasubramanyam A et al. Glutathione synthesis is diminished in patients with uncontrolled diabetes and restored by dietary supplementation with cysteine and glycine. Diabetes Care 2011; 34: 162–167. 20929994 10.2337/dc10-1006 PMC3005481

